# The usefulness of a new rapid diagnostic test, the First Response^® ^Malaria Combo (pLDH/HRP2) card test, for malaria diagnosis in the forested belt of central India

**DOI:** 10.1186/1475-2875-7-126

**Published:** 2008-07-11

**Authors:** Praveen K Bharti, Nipun Silawat, Pushpendra P Singh, Mrigendra P Singh, Manmohan Shukla, Gyan Chand, Aditya P Dash, Neeru Singh

**Affiliations:** 1 National Institute of Malaria Research, Field Station, Jabalpur, Madhya Pradesh, India; 2 Regional Medical Research Centre for Tribals, Jabalpur, Madhya Pradesh, India; 3 National Institute of Malaria Research, Delhi, India

## Abstract

**Background:**

Malaria presents a diagnostic challenge in tribal belt of central India where two Plasmodium species, *Plasmodium falciparum *and *Plasmodium vivax*, are prevalent. In these areas, rapid detection of the malaria parasites and early treatment of infection remain the most important goals of disease management. Therefore, the usefulness of a new rapid diagnostic (RDT), the First Response^® ^Combo Malaria Ag (pLDH/HRP2) card test was assessed for differential diagnosis between *P. falciparum *with other Plasmodium species in remote villages of Jabalpur district.

**Methods:**

A finger prick blood sample was collected to prepare blood smear and for testing with the RDT after taking informed consent. The figures for sensitivity, specificity, accuracy and predictive values were calculated using microscopy as gold standard.

**Results:**

Analysis revealed that overall, the RDT was 93% sensitive, 85% specific with a positive predictive value (PPV) of 79%, and a negative predictive value (NPV) of 95%. The accuracy 88% and J-index was 0.74. For *P. falciparum*, the sensitivity and specificity of the test were 96% and 95% respectively, with a PPV of 85% and a NPV of 99%. The RDT accuracy 95% and J-index was 0.84. For non-falciparum malaria, the sensitivity, specificity and accuracy were 83%, 94% and 92% respectively with a PPV of 69% and a NPV of 97%.

**Conclusion:**

The RDTs are easy to use, reliable and simple to interpret. RDTs are more suited to health workers in situations where health services are deficient or absent. Therefore, the test can be used as an epidemiological tool for the rapid screening of malaria.

## Background

Malaria is a major public health problem in tribal belt of Central India where only two Plasmodium species, i.e. *Plasmodium falciparum *and *Plasmodium vivax *are prevalent [1,2]. The ethnic tribes that live in these areas often travel several hours or days to reach the nearest Primary Health Centre (PHC). In such areas laboratory facilities for diagnosis of malaria are often not available and the clinical signs alone can not identify patients with malaria. Diagnosis of malaria made on the basis of clinical symptoms is at best 50% accurate [3]. Further, PHC's clinics examining blood smears from a large number of clinically suspected patients are often limited by one or two trained microscopists resulting in misleading interpretation and underestimation of malaria parasites. Consequently, a considerable proportion of drugs have been wasted on patients with non malarial disease due to lack of prompt and accurate laboratory diagnosis. Presumptive treatment of malaria encourages the development and spread of drug resistant *P. falciparum *parasites [4]. Early diagnosis and prompt treatment (EDPT) of malaria with efficient drugs is required for effective malaria control.

Several rapid diagnostic test (RDTs) kits for malaria exist for situations in which reliable microscopy may not be available [5,6]. These tests are based on the detection of antigens released from parasitized red blood cells [7]. In the case of *P. falciparum*, these RDTs are based on detection of the *P. falciparum *histidine rich protein 2 (HRP2) or of the Plasmodium specific lactate dehydrogenase (pLDH). Species specific pLDH isoforms have been used to develop a test for *P. vivax *[8]. Recently another rapid test First Response^® ^Combo Malaria Ag (pLDH/HRP2) card test was developed in India for differential diagnosis between *P. falciparum *and the other plasmodium species. To determine the usefulness of new rapid test in low endemic area where both *P. falciparum *and *P. vivax *are prevalent, the diagnostic capacity of First Response^® ^Combo Malaria Ag (pLDH/HRP2) card test (Premier Medical Corporation Ltd., Mumbai, India) was compared with that of expert microscopy, the gold standard. Additionally, the ease of use and accuracy of the test was also assessed.

## Methods

### Study area

Jabalpur district in central India has a mixed rural, urban and tribal population. This work was performed in Bargi PHC located in forest in 25 km radius, from 21 August – 30 September 2007 during peak monsoon season. The terrain of study area is highly undulating and inaccessible. Villages are remote, thinly populated formed of six to 10 hamlets and located in field and forest. The inhabitants are mainly ethnic Gond tribe (60% – 80%) and agriculture is monsoon-dependant. Inhabitants are poor and live in small, dark, mud plastered huts without electricity. During the rains, perennial streams and its tributaries creates small water pool and remain as potential breeding site for several months in which both the malaria vector, *Anopheles culicifacies *and *Anopheles fluviatilis *breed profusely. Medical facilities are non-existent.

### Sample collection

All fever cases in ten villages were screened for malaria independently by microscopy and RDT to evaluate the performance of the test under field conditions. Make-shift field clinics were established in field where persons of all ages visit for checkup, thus permitting performance of the RDT in all persons suspected to have malaria, whatever their history (recent malaria attack or with a history of malaria in the previous 15 days), clinical status (high or low grade fever, severe or mild symptoms) and other factors that may affect the sensitivity and the specificity of the RDT. A questionnaire was filled for each patient with basic clinical and demographic information after taking verbal consent. The RDT kits were opened only after the patient had been selected and interviewed by the medical staff. Blood was obtained by finger prick for the First Response^® ^Combo Malaria Ag (pLDH/HRP2) card test and thick smear before patients received treatment. In all 291 cases were tested by the RDT after taking verbal consent.

### RDT interpretation

The First Response ^®^Malaria pLDH/HRP2 Combo test contains a membrane strip, which is pre-coated with two monoclonal antibodies as two separate lines across a test strip. One monoclonal antibody (test line 2) is pan-specific to lactate dehydrogenase (pLDH) of the *Plasmodium *species (*P. falciparum, vivax, malariae, ovale*) and the other line (test line 1) consists of a monoclonal antibody specific to histidine-rich protein 2 (HRP2) of the *P. falciparum *species. The conjugate pad is dispensed with monoclonal antibodies, which are pan-specific to pLDH and *P. falciparum *specific to HRP2. Blood sample was measured in a calibrated dropper capable of delivering 5 μl sample accurately into sample well followed by two drops of assay buffer (60 μl) into developer well. Test card has one control line to indicate the validity of the test procedure and it's working condition. Control and test lines appeared within 20 minutes in a reading window. Thus, the RDT is designed for the differential diagnosis between *P. falciparum *and other *Plasmodium *species. The interpretation of the test is as described below:

#### *Plasmodium falciparum *positive reaction

The presence of three bands (control, test line 2 and test line 1) or two bands (control and test line 1) indicates a positive result for *P. falciparum *(or *P. falciparum *plus other non-falciparum species).

#### *Plasmodium vivax *or other *Plasmodium *species positive reaction

The presence of two bands (control and test line 2) indicates a positive result for non-falciparum malaria. The pLDH present in the sample reacts with the pan anti-pLDH conjugate and moves through the test strip where the pLDH is captured by pan specific anti-pLDH.

#### Negative reaction

The presence of only one band in the control area indicates a negative result. A one-hour workshop, including training in blood collection from finger prick, performance and interpretation of RDT was conducted at National Institute of Malaria Research Field Station Jabalpur (NIMR) under the Indian Council of Medical Research (ICMR) laboratory by one Medical Officer to two Field Laboratory Assistants (FLAs). All specimens were tested on site with the RDT by the FLAs as per manufacturer's instructions. Simultaneously, thick blood smears were also prepared.

### Blood smears and microscopy

The blood smears were stained with JSB stain [9] and examined on the same day by an experienced microscopist in the laboratory of NIMR, without reference to the results of the RDT/clinical status. Results of the RDT and microscopy examination were recorded on separate sheets. The microscopist examined 100 microscopic field of thick smear before classifying a smear as negative. Parasite densities were calculated according to the standard method (parasite/μl= no. of asexual parasites × 8,000/no. of WBC counted) [10]. The result of both microscopy and RDT were matched by an independent expert who was blinded to the patient's clinical status, microscopy and RDT results.

### Treatment

All patients infected with *P. falciparum *and *P. vivax *were given standard treatment as per National Vector-Borne Disease Control Programme (NVBDCP). All adult subjects with *P. falciparum *were administrated the standard oral dose of chloroquine (1,500 mg chloroquine in three days) followed by primaquine (45 mg as a single dose). Non-falciparum cases were given 1,500 mg chloroquine in three days, followed by 15 mg primaquine daily for five days. Infants and children were given proportionally lower doses. Infants were not given primaquine as per National Vector Borne Disease Control Programme.

### Quality control

If the results of the RDT testing conflicted with that of the microscopy for any sample, the blood smear was re-examined by a different technician. This microscopist was also blinded to the previous microscopy and RDT results. If this re-examination gave a different result to the first examination, the second result was confirmed by a third examination by another technician.

Each RDT was saved as documentation for future reference. An independent staff re-read the saved tests after two months and matched with that original interpretation of results. The RDTs were stored properly (temperature 4 – 30°C) and used within shelf life. Only tests from one batch were used (Manufacture June 07, expiry January 09 batch no. 61F0107).

### Data analysis

The performance of RDT was expressed by calculating the sensitivity, specificity, positive predictive values (PPV) and negative predictive values (NPV) for *P. falciparum *and non falciparum malaria separately taking microscopy results as gold standard. The figures for specificity, sensitivity, predictive values and efficiency were calculated as suggested by Tjitra *et al *[11]. Data were double entered, validated and analysed using Epi Info™ 3.3.2 software (CDC Atlanta GA, USA). Proportions were compared using the chi-square test. The study protocol was approved by the ethics committee of the NIMR, Delhi.

## Results

In all 291 patients (M:F, 1:1.15) with fever were suspected of having malaria (age range < 1–60 years). The mean duration of fever was four days (range 1 – 20 days) and mean temperature was 100.5 ± 1.0 (range 98.6 – 103°F) while in malaria infected persons mean temperature was 100.9 ± 1.1 (range 99 – 103°F). Out of 291, 113 (39%) were found malaria infected, 41 with *P. vivax *(14%), 71 with *P. falciparum *which also include one mixed infection (25%). Table 1 shows a breakdown of malaria cases in different age groups.

**Table 1:** Age group-wise prevalence of malaria among symptomatic patients (August – September 2007)

Age Groups (yrs)	BSE*	Positive	*P. vivax*	*P. falciparum*	SPR†	SfR‡	Pf%§
≤1	7	2	1	1	28.6	14.3	50.0
> 1 through 4	26	8	6	2	30.8	7.7	25.0
> 4 through 8	44	25	10	15	56.8	34.1	60.0
> 8 through 14	57	19	8	11	33.3	19.3	57.9
> 14	157	59	16	43	37.6	27.4	72.9

Total	291	113	41	72	38.8	24.7	63.7

* Blood Slide Examined

† Slide Positivity Rate (number of parasitaemic cases per 100 examined slides)

‡ Slide falciparum Rate (number of falciparum cases per 100 examined slides)

§*Plasmodium falciparum *percentage (number of falciparum cases per 100 parasitaemic cases)

The results of parasite detection by microscopy and RDT were compared in Table 2. Microscopically-confirmed *P. falciparum *were 72, of which RDT detected 69 matching positives. The asexual parasitaemias ranged from 80 -111,920 parasites/μl (mean ± sd 8010.5 ± 21595.2). Only three subjects were found as false negatives and 12 as false positives. The sensitivity and specificity of the test for *P. falciparum *was 96% and 95% respectively. The PPV and NPV were 85% and 99% respectively. The accuracy was 95% and J index 0.84 (Table 3). Only two subject positive for *P. falciparum *by microscopy with very low parasite density (120 parasites/μl) were tested positive as non-falciparum malaria by RDT.

**Table 2:** Diagnostic performance of First Response^® ^Malaria Ag (pLDH/HRP2) card test Vs Light Microscopy as reference standard

Microscopy		First Response^® ^Malaria Ag (pLDH/HRP2) card test		
Results	N (%)	Negative	*P. falciparum*	Non *P. falciparum*
Negative	178 (61.2)	153	12	13
*P. falciparum*	72 (24.7)	1	69	2
Non-*P. falciparum*	41 (14.1)	7	0	34

**Table 3:** Sensitivity, specificity and accuracy of First Response^® ^Malaria Ag (pLDH/HRP2) card test by Light Microscopy

Indices	Overall	*P. falciparum*	Non-*P. falciparum *species
True Positive	103	69	34
True Negative	153	207	235
False Positive	27	12	15
False Negative	8	3	7
Sensitivity (95% CI)	93 (86–96)	96 (88–99)	83 (69–91)
Specificity (95% CI)	85 (79–89)	95 (91–97)	94 (90–96)
PPV (95% CI)	79 (71–85)	85 (76–91)	69 (55–80)
NPV (95% CI)	95 (91–97)	99 (96–99)	97 (94–99)
Accuracy (95% CI)	88 (83–91)	95 (92–97)	92 (87–95)
J-index	0.74	0.84	0.67

Out of 41 non-falciparum infections, RDT detected 34 matching positives, seven false negatives and 15 false positives. The asexual parasitaemias ranged from 200 – 14,800 parasites/μl (mean ± sd 1,871.58 ± 33,64.43). The sensitivity of the test for non-falciparum malaria 83% which was significantly lower when compared with *P. falciparum *(≤ 0.05). However, specificity (94%), accuracy (92%), PPV (69%) and NPV (97%) were not significantly different from the corresponding values for *P. falciparum*.

Overall (pooled *P. falciparum *and non falciparum infections), the sensitivity and specificity were 93% and 85% respectively with a PPV of 79% and a NPV of 95%. A comparison of parasitaemia versus RDT sensitivity showed that with parasitaemia of ≥ 120 parasites/μl, RDT was 98% sensitive for *P. falciparum*. The only exception was one subject with the parasite count of 840 parasites/μl which was negative by RDT. For non-falciparum infections, the RDT did not identify seven subjects out of 41, some of these, but not all had low parasitaemia (≤ 500 parasites/μl) and one subject with a parasite count of 4,480 parasites/μl.

The test was evaluated as very easy to perform, as the sampling pipette made it very easy to measure exact 5 μl of blood to be dispensed onto the sample well. The cassettes were simpler to use and this is likely to affect test accuracy. The results did not change after the 20 minutes. These RDTs were reread after two months and the results matched with that of original results.

## Discussion

Several RDTs for malaria exist, which are fast, easy to perform and can be carried out by unskilled staff [7,12]. Of these, two RDT, ParaHIT *f *[13] and Paracheck-Pf [14] based on the detection of parasite HRP2 have proven superior to other tests [15,16]. Recently introduced First Response RDT was evaluated for diagnostic capacity in central India in an area of Jabalpur where malaria morbidity is rising [17], especially *P. falciparum *due to labour migration and other technical/administrative factors [14].

Results indicate that sensitivity for non falciparum malaria infections in this study is markedly lower (83%; CI, 69–91) than the corresponding values for *P. falciparum *(96%; CI, 88–99), which is consistent with other studies[8,18,19]. It's known that anti-pLDH antibodies are likely to be less temperature stable than HRP2 specific antibodies and looses sensitivity more rapidly in uncontrolled storages[20]. Further, high humidity can rapidly degrade pLDH based RDTs. This study was carried out during main rainy season when humidity ranged between 80–100%. However, > 80% sensitivity recorded in this study is relatively better than the ICT Pf/Pv RDT tested earlier for non-falciparum infections (72% sensitivity) in central India [6]. The NPVs were high for both *P. falciparum *and non-falciparum malaria. In field setting, a negative test corresponds in the vast majority of cases to a non-infected individual.

However, from a clinical perspective, failure to diagnose *P. falciparum *at 840 parasites/μl or non falciparum malaria at 4,480 parasites/μl is a serious cause of concern. This can be potentially dangerous, to miss the diagnosis of malaria in a febrile patient which may develop complications in the absence of appropriate treatment. False negative RDT results in samples with higher parasitaemia have been reported in earlier studies but the underlying reasons are not known [19,21,22]. Polymorphisms in the Pf HRP2 protein may explain some of the variability in RDT performance as extensive diversity was observed in Pf HRP2 sequences [23]. Further, the possibility of coincidental rheumatoid factor causing false positive results can not be excluded [24,25]. Among non-falciparum infections, *P. vivax *is no longer considered a mild infection [26-28]. Analysis revealed a relatively large number of non falciparum infections false positive too. The reasons for the false positivity of RDTs for non falciparum infections are unknown. It is likely that some of the false-positive cases were true positives which were not detected by microscopy due to very low parasitaemia. However, these are unlikely to be applicable for the entire set of false positive cases. It is probable that some of our patients with false positive results may have taken self medication with antimalarial drugs during an attack of fever as prior self-medication with antimalarials could not be completely excluded. Thus in areas of low and moderate malaria transmission, rapid tests require a high sensitivity at lower densities of infection, to serve the non immune populations that can suffer from clinical disease at much lower parasitaemia as opposed to people in high endemic areas in Africa [29]. Clearly, more accurate results would be expected if PCR had been used as the reference standard, since PCR based methodology detect parasitaemia below the limits of microscopy [18]. Further studies are required to test this RDT in a group of patients with known/proven arthritis, connective tissue disorder, tuberculosis, typhoid/salmonella infection etc to confirm the extent of cross reactivity.

The two microscopically detected *P. falciparum *case shown as non-falciparum infections by RDT could have been due to mixed infections with non-falciparum parasites. Further studies in various epidemiological settings are required to establish accurately performance characteristics of this new test.

In addition to performance of this RDT, some operational observations were also made. The RDTs were re-read later and recorded that results were not changed as recorded earlier using MAKROmed RDTs in South Africa [30].

In remote and resource poor areas of central India microscopy is not readily available and it can take -four to six weeks before blood smear results are available as materials, supply lines, trained staff are not sufficient. Additionally, daily power cuts for- four to six hours is a major problem. The delay in the diagnosis and treatment of cases contributes to the continuing transmission. To control malaria, programme managers have to depend on EDPT[31]. Given the logistic and financial difficulties of microscopy in most field settings, only RDTs are viable option at the present time in such areas.

However, despite its advantages over microscopy and clinical diagnosis, the cost of this RDT is high $1.15 per test (Nilesh Mehta, CEO & President PMC, Mumbai, personal communication) and prevents its wide-spread use in malaria endemic areas of developing countries where many patients need a fever screen. Commercial interest in producing RDTs at a cost that many of the tropical countries could afford is a subject of ongoing debate. However, whatever the RDT costs, the cost-effectiveness of the accurate diagnosis of malaria will become apparent as cheap drug CQ may no longer be effective. Furthermore, RDTs in cassette format tend to be simpler to use and this is likely to affect test accuracy and may provide saving through improved diagnosis.

In conclusion, the test is reliable and simple to interpret. The test is a potential alternative to microscopy in places where the facilities for microscopy are poor. Therefore, it is reasonable to consider future use of RDTs as an epidemiological tool for the rapid screening of malaria.

## Authors' contributions

PKB: Data collection, analysis and interpretation of results. NS: Data collection. PPS: Data collection. MPS: Statistical analysis and interpretation of results. MS: Clinical assessment and treatment of patients. GC: Data collection. APD: Coordination of the study and manuscript preparation. NS*: Study design, preparation of manuscript, and critically reading the manuscript for intellectual content. All authors read and approved the final manuscript.
